# Outcomes, Measurement Instruments, and Their Validity Evidence in Randomized Controlled Trials on Virtual, Augmented, and Mixed Reality in Undergraduate Medical Education: Systematic Mapping Review

**DOI:** 10.2196/29594

**Published:** 2022-04-13

**Authors:** Lorainne Tudor Car, Bhone Myint Kyaw, Andrew Teo, Tatiana Erlikh Fox, Sunitha Vimalesvaran, Christian Apfelbacher, Sandra Kemp, Niels Chavannes

**Affiliations:** 1 Lee Kong Chian School of Medicine Nanyang Technological University Singapore Singapore; 2 Department of Primary Care and Public Health School of Public Health Imperial College London London United Kingdom; 3 Centre for Population Health Sciences, Lee Kong Chian School of Medicine Nanyang Technological University Singapore Singapore; 4 Department of Internal Medicine Onze Lieve Vrouwen Gasthuis Amsterdam Netherlands; 5 Institute of Social Medicine and Health Systems Research Otto von Guericke University Magdeburg Magdegurg Germany; 6 Family Medicine and Primary Care Lee Kong Chian School of Medicine Nanyang Technological University Singapore Singapore; 7 Faculty of Health Sciences Curtin Medical School Curtin University Bentley Australia; 8 Department of Public Health and Primary Care Leiden University Medical Centre Leiden Netherlands

**Keywords:** virtual reality, augmented reality, mixed reality, outcomes, extended reality, digital education, randomized controlled trials, medical education, measurement instruments

## Abstract

**Background:**

Extended reality, which encompasses virtual reality (VR), augmented reality (AR), and mixed reality (MR), is increasingly used in medical education. Studies assessing the effectiveness of these new educational modalities should measure relevant outcomes using outcome measurement tools with validity evidence.

**Objective:**

Our aim is to determine the choice of outcomes, measurement instruments, and the use of measurement instruments with validity evidence in randomized controlled trials (RCTs) on the effectiveness of VR, AR, and MR in medical student education.

**Methods:**

We conducted a systematic mapping review. We searched 7 major bibliographic databases from January 1990 to April 2020, and 2 reviewers screened the citations and extracted data independently from the included studies. We report our findings in line with the PRISMA (Preferred Reporting Items for Systematic Reviews and Meta-Analyses) guidelines.

**Results:**

Of the 126 retrieved RCTs, 115 (91.3%) were on VR and 11 (8.7%) were on AR. No RCT on MR in medical student education was found. Of the 115 studies on VR, 64 (55.6%) were on VR simulators, 30 (26.1%) on screen-based VR, 9 (7.8%) on VR patient simulations, and 12 (10.4%) on VR serious games. Most studies reported only a single outcome and immediate postintervention assessment data. Skills outcome was the most common outcome reported in studies on VR simulators (97%), VR patient simulations (100%), and AR (73%). Knowledge was the most common outcome reported in studies on screen-based VR (80%) and VR serious games (58%). Less common outcomes included participants’ attitudes, satisfaction, cognitive or mental load, learning efficacy, engagement or self-efficacy beliefs, emotional state, competency developed, and patient outcomes. At least one form of validity evidence was found in approximately half of the studies on VR simulators (55%), VR patient simulations (56%), VR serious games (58%), and AR (55%) and in a quarter of the studies on screen-based VR (27%). Most studies used assessment methods that were implemented in a nondigital format, such as paper-based written exercises or in-person assessments where examiners observed performance (72%).

**Conclusions:**

RCTs on VR and AR in medical education report a restricted range of outcomes, mostly skills and knowledge. The studies largely report immediate postintervention outcome data and use assessment methods that are in a nondigital format. Future RCTs should include a broader set of outcomes, report on the validity evidence of the measurement instruments used, and explore the use of assessments that are implemented digitally.

## Introduction

### Background

Extended reality (ER) encompasses immersive technologies within the reality-virtuality continuum, such as virtual reality (VR), augmented reality (AR), and mixed reality (MR). The use of ER technologies is becoming more common in medical education. These technologies offer a wide range of educational opportunities within different medical specialties. VR is a technology that renders a fully computer-generated 3D multimedia environment in real time. It supports a first-person active-learning experience through immersion, that is, a perception of the digital world as real. VR can be integrated with other educational approaches such as virtual patients or serious games. VR patient simulations are interactive computer simulations of real-life clinical scenarios for the purpose of medical education. VR serious games incorporate gaming concepts such as different levels of difficulties, rewards, or feedback within the computer-generated 3D environment.

AR is a technology in which the real-world environment is enhanced by computer-generated virtual imagery information. In AR, virtual objects are projected over the real-world environment. MR is a hybrid technology that merges the features of VR and AR. In MR, virtual objects become a part of the real word. ER technologies can be displayed through desktop computers, mobile devices, and large screens or projected on the walls. They can be purely screen based or also involve the use of joysticks, probes, gloves, simulators, and other forms of haptic devices.

### Effectiveness of VR

Our systematic review on the effectiveness of VR for health professions education showed that VR may improve postintervention knowledge and skills outcomes compared with traditional education (ie, nondigital education) or other types of digital education such as online or offline digital education [1]. Data for other outcomes were limited. Systematic reviews of randomized controlled trials (RCTs) remain the gold standard for evidence on the effectiveness of interventions. However, the heterogeneity of participants, interventions, comparison interventions, and outcomes reported in the individual studies can limit the trustworthiness of the systematic review findings and preclude a meta-analysis. Similarly, differences in measurement instruments and types of validity evidence can lead to unreliable conclusions [2]. The choice of digital education outcomes can be influenced by different factors, including types of digital education, the curriculum, and the field of study [3,4]. The process of measuring digital education outcomes can be achieved with a wide variety of measurement instruments, including multiple-choice questions, structured essays, and structured direct observations with checklists for ratings [5]. Measurement instruments used in research need to have validity evidence. Validity is defined as “the degree to which evidence and theory support the interpretations of test scores entailed by the proposed uses of tests” [6]. Validity evidence for measurement instruments is important to ensure that the instruments reliably measure what they purport to measure and to support the interpretation of assessment data. However, reporting of validity evidence of measurement instruments in health professions education literature is still suboptimal, ranging from 34.6% in studies on continuing medical education to 64% in studies on technology-enhanced health professions simulation training [7,8].

The use of measurement instruments without validity evidence severely undermines the credibility of the research results [9]. ER is increasingly used in medical education, and studies in this field should evaluate diverse outcomes using outcome measurement instruments with validity evidence. Our aim is to support this by mapping the current choice of outcomes, measurement instruments, and the prevalence of measurement instruments with validity evidence in RCTs on the use of ER in undergraduate and preregistration medical education.

## Methods

### Methodology, Definitions, and Eligibility Criteria

We performed this systematic review in line with the Cochrane gold standard systematic review methodology and report it according to the PRISMA (Preferred Reporting Items for Systematic Reviews and Meta-Analyses) standards of quality for reporting systematic reviews [10,11]. In this review, we aim to answer the following research questions:

1. Which outcomes (eg, knowledge, skills, attitudes, and behavior) are assessed and reported in RCTs on the effectiveness of VR, AR, and MR in undergraduate and preregistration medical education?
2. What type of measurement instruments were used in RCTs on the use of VR, AR, and MR in undergraduate and preregistration medical education?
3. What proportion of RCTs on the use of VR, AR, and MR in undergraduate medical education report validity evidence for the measurement instruments used, and how was the evidence reported?

We included studies meeting the following eligibility criteria:

1. RCTs
2. Studies on students participating in preregistration or undergraduate medical education in any geographical or educational setting
3. Studies evaluating any type of blended (ie, a combination of extended and nondigital, traditional education) or full ER technology, including VR, AR, and MR
4. Studies comparing VR with control interventions such as classroom-based learning, no intervention, and other types of digital and blended education

We defined different ER technologies as per Textbox 1. Preregistration or undergraduate medical education was defined in line with the World Health Organization (WHO) definition as “any type of initial study leading to a qualification that (i) is recognized by the relevant governmental or professional bodies of the country where the study was conducted and (ii) enables its holder primary entry into the healthcare workforce” [12]. Studies were excluded if they focused on traditional and complementary medicine as defined by WHO (as such education is not included in most medical schools) and used study designs other than an RCT [13].

**Descriptions and classification of different types of virtual reality (VR), augmented reality (AR), and mixed reality (MR).**

**Types of extended reality modalities in medical education**

- VR is a technology that allows the user to explore and manipulate computer-generated 2D or 3D, multimedia sensory environments in real time [14]. The VR environment is the computer-generated representation of a real or artificial environment that can be interacted with by external involvement, allowing for a first-person active-learning experience through immersion [15].
- Screen-based VR interventions are computer-based 3D software applications delivered either through computer screens or head-mounted displays (ie, VR headsets). This type of VR in medical education mostly includes 3D models of organs and VR worlds.
- VR simulators or psychomotor skills trainers encompass use of VR technology and physical probes or objects that help the learners to connect with the objects from the VR environment and convey feedback or tactile sensation to the learners.
- VR patient simulation refers to the interactive computer simulations of real-life clinical scenarios in VR for the purpose of medical training, education, or assessment [16]. They include virtual patients represented by computer-generated 2D or 3D characters or avatars.
- VR serious gaming or gamification intervention involves gaming concepts such as different levels of difficulties, rewards, feedback, and so on, within the computer-generated VR environment for learning purposes.
- AR is a technology that allows a live real-time direct or indirect real-world environment to be augmented or enhanced by computer-generated virtual imagery information (eg, smart, virtually enhanced glasses). Computer-generated information is overlaid on the real-world environment. AR is distinct from VR in which only a computer-generated image is supplied to the user [17].
- MR is a hybrid technology that merges the features of VR and AR [18]. In MR, physical and virtual or digital objects are displayed together and the features of *virtuality* and reality are merged for the learners [19].

### Electronic Searches

We developed a comprehensive search strategy for MEDLINE (Ovid), Embase (Elsevier), Cochrane Central Register of Controlled Trials (Wiley), PsycINFO (Ovid), Education Resources Information Center (Ovid), CINAHL (EBSCO), and Web of Science Core Collection (Thomson Reuters). Databases were searched from January 1990 until April 2020 without language restrictions.

We used 1990 as the starting year for our search because before 1990, the use of computers was uncommon for educational use. We used the MEDLINE strategy presented in Multimedia Appendix 1. This was adapted to search the other databases with the help of a librarian (Ms Yasmin Munro). To identify unpublished studies, we searched the International Clinical Trials Registry Platform Search Portal and metaRegister of Controlled Trials. We also checked reference lists of relevant systematic reviews and potentially eligible studies against the inclusion criteria.

Search results across different databases were compiled using EndNote X8 software (Clarivate), and duplicate records were removed. In all, two pairs of two reviewers (BMK, AT, TEF, and SV) independently screened the studies, extracted the data, and carried out data analysis. Any disagreements were resolved by a discussion between the 2 reviewers, with a third reviewer acting as an arbiter if needed. The PRISMA flow diagram was used to report the selection and inclusion of studies [10].

### Data Extraction

The data for each of the included studies were independently extracted and managed by 2 reviewers using a structured data recording form, which included information about the study characteristics such as reference of the study, country of the study, the WHO region of the study, name of measurement instrument, description of measurement instrument, types of outcomes reported, assessment category of measurement instrument [5], assessment method of measurement instrument, types of participants, sample size, raters of the instrument, procedure of identifying the raters, and training of the raters for the instruments [20]. We recorded all information relating to validity evidence sources and measurement properties that were reported directly in the articles [5,6]. We also recorded any validity evidence recorded indirectly; for example, through a reference to a validation study focusing on a particular measurement instrument. If the studies presented more than one outcome measure, relevant details of the second outcome measure were also recorded. The data extraction form was piloted and amended according to feedback received. We contacted the study authors for further data in case of missing information.

### Data Analysis and Synthesis

We analyzed and synthesized the data as follows: (1) we ascertained the types of primary and secondary outcome measurement instruments; (2) we classified and mapped the data according to types of outcomes (eg, knowledge, skills, attitudes, satisfaction, or competencies); intervention (eg, VR vs classroom-based learning and VR vs serious gaming); year of medical studies (ie, first year, second year, or final year), types of measurement instruments (eg, written exercises [surveys with only multiple-choice questions and surveys with other types of questions and essays] vs in-person assessment where an examiner observed performance [eg, global ratings, structured direct observation, and objective structured clinical examinations]); assessment delivery mode (ie, digital vs classroom-based assessment); and discipline (eg, laparoscopic surgery, anatomy, and internal medicine); and (3) we determined the proportion of RCTs on the use of VR, AR, and MR in undergraduate medical education using measurement instruments with sufficient validity evidence in relation to the goal of the measurements (*validity evidence*). The aim of this study is to comprehensively document outcomes and measurement instruments rather than to synthesize data about the effect of the interventions [6]. Therefore, we did not undertake a risk-of-bias assessment of the studies because it was not relevant to the objectives of this review.

We assessed the validity evidence of the measurement instruments as reported in the cited validation studies using the Consensus-Based Standards for the Selection of Health Measurement Instruments (COSMIN) taxonomy of measurement properties [21]. The COSMIN taxonomy outlines three measurement properties or validity evidence domains: reliability, validity, and responsiveness. The reliability domain encompasses measurement properties such as internal consistency, reliability, and measurement error. The domain validity contains the measurement properties such as content validity (including face validity), construct validity (including structural validity, hypotheses testing, and cross-cultural validity and measurement invariance), and criterion validity [21].

Digital assessments were defined as assessments that were delivered exclusively using digital technology (ie, PCs, laptops, mobile phones, and tablets) and included online surveys, questionnaires, computer scoring, or the use of software metrics such as time to completion, number of errors, path length, and so on. Assessments in which digital tools (eg, video recordings or Microsoft PowerPoint presentations) were used to facilitate classroom-based assessment, such as written exercises or in-person observation by the examiners, were not categorized as digital assessments.

### Ethics Approval

This systematic mapping review is an analysis of published studies and as such, did not require an ethics approval.

## Results

### Study Characteristics

**Figure 1:**
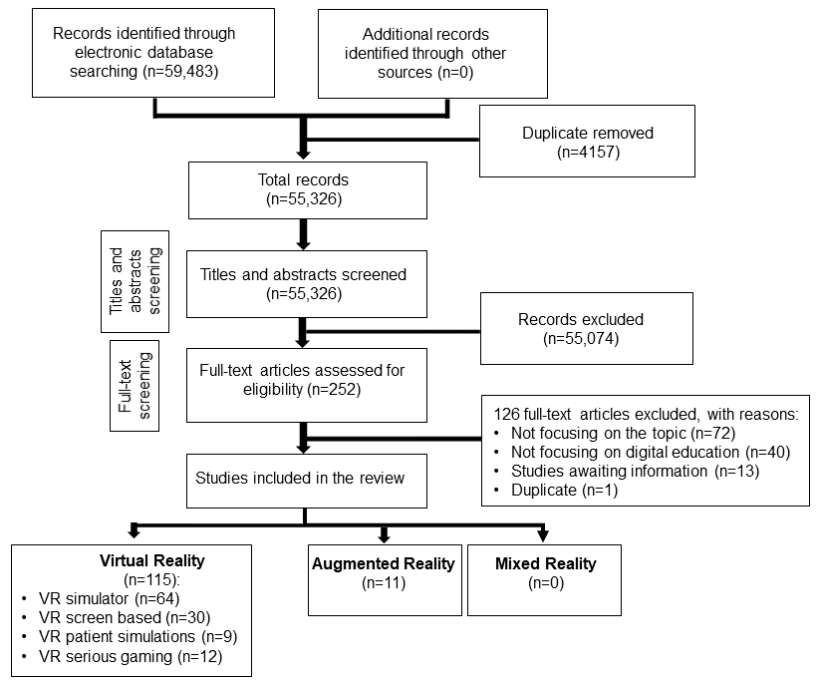
Study flow diagram. VR: virtual reality.

The searches identified 59,483 records through electronic databases, of which we included 126 (0.21%) RCTs. Of the 126 RCTS, 115 (91.3%) assessed different forms of VR, whereas 11 (8.7%) focused on AR simulations (Figure 1). We did not find any study evaluating the use of MR in medical student education.

Of the 115 included articles focusing on VR-based training for medical student education, 64 (55.7%) focused on VR-based psychomotor skills training [22-85], 30 (26.1%) on screen-based VR [86-115], 9 (7.8%) on VR patient simulations [116-124], and 12 (10.4%) on VR serious gaming and gamification [125-136]. Only 8.7% (11/126) of the included studies focused on AR simulations [137-147] and none focused on MR training in medical student education. The included studies were published between 1997 and 2020. Most of the studies were from high-income countries, except for 8.7% (11/126) of the studies, which were conducted in low- and middle-income countries [35,36,72,75,105,114,126,127,132,134,139]. Of the 126 studies, 31 (24.6%) cited validation studies for the measurement instruments used [23,25,27,30-32,34-36,47,48, 52,58,60,63-65,70,72,78,79,82,84,92,101,118-120,126,128,133] (Multimedia Appendices 2 and 3).

Participants included medical students from the first to sixth year of medical schools (N=9010). The studies compared the use of VR and AR training (either stand-alone intervention or blended with traditional, nondigital learning) with traditional, nondigital learning or a different form of VR and AR training or other forms of digital education such as online digital education or offline digital education. Of the 64 studies focused on the effects of VR simulators for medical student education, 61 (95%) were delivered in a university setting, whereas 3 (5%) were conducted in a hospital setting [37,72,74].

### VR Simulators

Of the 115 VR studies, 64 (55.6%) with 3132 medical students evaluated the effects of VR simulators in medical student education [22-85]. The studies included first year to sixth year medical students and were published between 2001 and 2020. In terms of the topic or area of study, 53% (34/64) of the studies focused on laparoscopic surgery [22,24,27,31,35-38,40, 41,45-48,50-52,54,56-66,69,78,81-83]; 16% (10/64) on surgery [25,28,53,55,67,68,71,74,76,77]; 8% (5/64) on orthopedic surgery [39,42,73,79,84]; 8% (5/64) on ureteroscopy [30,33,34,80,85]; 5% (3/64) each on ophthalmology [26,70,75] and intravenous cannulation [29,32,72], and 2% (1/64) each on endoscopy [49], colonoscopy [23], shoulder-joint clinical anatomy [44], and empathic communication skills [43].

**Figure 2:**
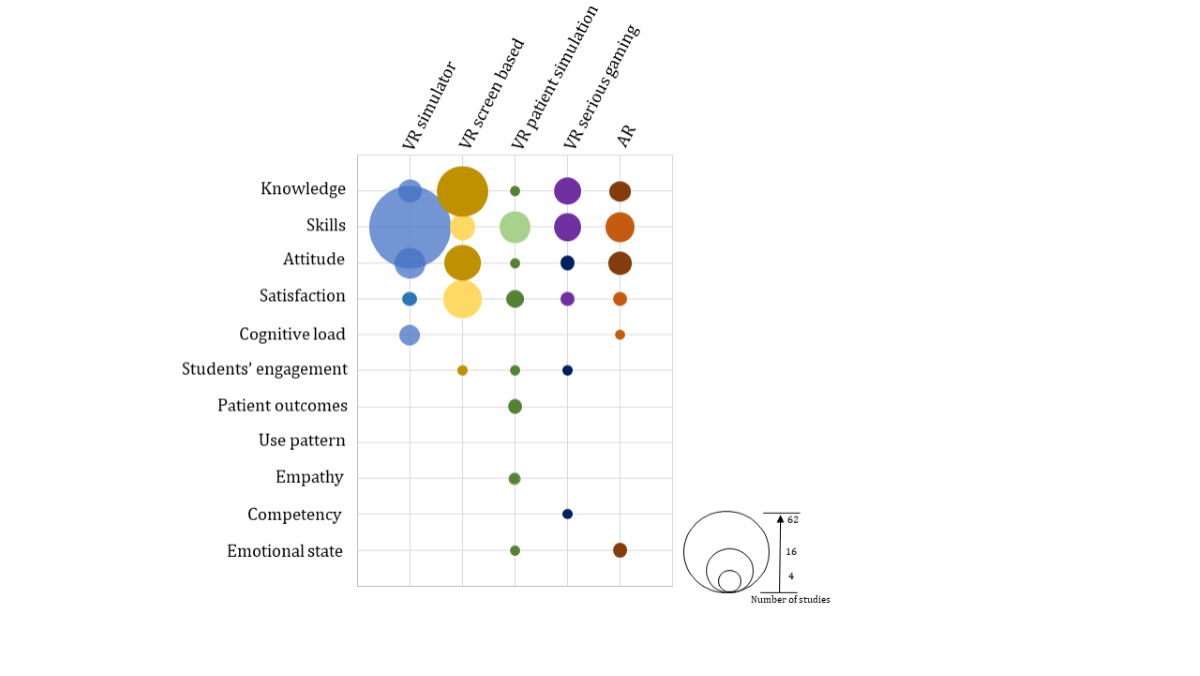
Types of reported outcomes in virtual reality (VR)– and augmented reality (AR)–based training.

For the outcomes, 97% (62/64) of the studies reported on participants’ postintervention skills [22-43,45-53,55-85], 8% (5/64) on knowledge [28,37,44,54,65], 14% (9/64) on attitudes toward the intervention [31,32,44,48,54,65,66,71,75], 3% (2/64) on satisfaction [68,71] and 6% (4/64) on cognitive load [25,27,39,63](Figure 2). Of the 62 studies that reported on participants’ postintervention skills, 11 (18%) reported change score from baseline for the skills outcome [25,50,56,58,68,73,76-78,80,85] and 1 (2%) reported change score from baseline for the satisfaction outcome [68]. Regarding retention, 7.8% (5/64) of the studies assessed skills retention at 2-4 weeks after the intervention [25,31,33,40,83]. The remaining studies did not report retention outcomes.

For modes of assessment, 46.8% (30/64) of the studies used paper-based written assessments or in-person assessments (ie, nondigital) using checklists by the examiners [24,25,31-37,39, 46,47,51-55,58,60,65,68,70-72,75,79-82,84]; 31% (20/64) used digital assessments such as software-based metrics (eg, time spent on training, number of errors, total path length, motion analysis, or checklists) [22,23,26,29,40-42,45,49,50,56,57,59, 61,62,64,69,73,78,85]; 11% (7/64) used a combination of digital assessments using software-based metrics, paper-based written assessments, or in-person assessments by supervising examiners [27,38,43,44,48,63,66] and 2% (1/64) used both paper-based written assessments and in-person assessments using checklists [37]. In 10.1% (7/64) of the studies, the mode of assessment was unspecified [28,30,67,74,76,77,83].

For validity evidence, 54.6% (35/64) of the studies reported a single form of validity evidence (mostly either internal consistency or reliability) for the measurement instruments largely used for assessment of skills [22,23,25,27,30-37,39,40, 47,48,51-55,58,60,63-66,68,70,72,78-80,82,84] (Multimedia Appendices 2 and 3). The remaining studies did not provide any information on the validity of assessment tools used for measuring the outcomes. Of the 64 studies, 23 (36%) referenced pertinent measurement instrument validation studies, largely used for assessment of skills (mostly either internal consistency or reliability) for the measurement instruments largely used for assessment of skills [23,25,27,30-32,34-36,47,48,52,58, 60,63-65,70,72,78,79,82,84]. Of the measurement properties, these studies mostly reported internal consistency and reliability, followed by structural validity and hypotheses testing.

### Screen-Based VR

Of the 115 VR studies, 30 (26.1%) studies with 2409 medical students evaluated the effect of screen-based or nontechnical training for medical students [86-115]. The studies included first year to sixth year medical students and were published between 1997 and 2020. In terms of the topic or area of study, 37% (11/30) of the studies focused on anatomy [87,91, 95-98,100,102,104,106,114]; 17% (5/30) on ophthalmology [93,109,112,113,115]; 17% (5/30) on surgery [88,90, 92,101,105]; 6% (2/30) each on patient examination [99,108] and one study each (3%,1/30) on operating room introduction [107], biomechanics of the spine [89], histology [111], trauma [94], traumatic head injury [86], radiology [103], and genetics [110].

For the outcomes, 80% (24/30) of the studies reported on participants’ postintervention knowledge [89,91,93-107, 109-115], 17% (5/30) on skills [88,92,99,101,107], 40% (12/30) on attitudes toward topics and interventions [86,87,90,91, 95,97,102-104,107,108,115], 47% (14/30) on satisfaction [87,89,91-93,97,98,100, 102,105,109,112-114] and 3% (1/30) on students’ learning engagement [89] (Figure 2). Of the 24 studies assessing knowledge, 5 (2%) also reported change score from baseline [101,104,105,113,114]. Similarly, 20% (1/5) of the studies assessing skills [101], 17% (2/12) of the studies assessing attitude toward the intervention [90,104], and 21% (3/14) of the studies assessing satisfaction [105,113,114] also reported change score from baseline. Regarding retention, only a single study assessed retention at 12 months after the intervention [112]. The remaining studies did not report outcomes at the follow-up stages.

Most of the studies (21/30, 70%) used paper-based written assessments [86,87,89-91,93,95,97,98,100,102-104,108-115]. Other forms of assessment included in-person assessments by an examiner [88], digital assessment in the form of questionnaires and ratings [94,105,106], combined paper-based written and in-person assessments [92,99,101,107], and a paper-based written assessment with questions delivered in the form of a PowerPoint presentation [96].

Of the 30 studies, 8 (27%) reported at least one form of validity evidence (mostly reliability) for the measurement instruments that were largely used to assess skills [88,91,92, 98,99,101,107,108]. Of these 8 studies, 2 (25%) referenced measurement instrument validation studies, both focusing on skills assessment and reporting on their reliability [92,101].

### VR Patient Simulations

Of the 115 VR studies, 9 (7.8%) with 782 medical students evaluated the effect of VR-based patient simulations in medical student education simulations [116-124]. Of these 9 studies, 4 (44%) focused on communication skills [117-119,124]; 2 (22%) on pediatric life support [121,122]; and 1 (11%) each on clinical reasoning [123], internal medicine [116], and suicide risk assessment [120] (Figure 2).

For the outcomes, 11% (1/9) of the studies reported on participants’ postintervention knowledge [122], 100% (9/9) on skills [116-124], 33% (3/9) on students’ satisfaction [119,120,123], 22% (2/9) on patient-related outcomes (eg, patients’ satisfaction) [119,120], and 11% (1/9) each on attitudes toward the intervention [124], engagement [123], mood changes or emotional state [124], and empathetic behavior [117]. None of the studies reported change score from baseline or retention data.

For mode of assessment, most of the studies used in-person assessments by an examiner [116-120,123,124] or paper-based written assessments [119,120,122,123]. Of the 9 studies, 2 (22%) used both paper-based written and in-person assessments by an examiner [119,120]; 1 (11%) used both digital assessments consisting of virtual patients and scoring and in-person assessment by an examiner [116]; and, finally, 1 (11%) used a combined assessment of digital assessment in the form of a survey, in-person assessment by an examiner, and paper-based written assessment for different outcomes [123].

Of the 9 studies, 5 (56%) reported at least one form of validity evidence (mostly internal consistency and reliability) for the measurement instruments used to assess skills [116-120] (Multimedia Appendices 2 and 3). Of these 5 studies, 3 (60%) referenced measurement instrument validation studies: 67% (2/3) focused on assessment of patient satisfaction [119,120] and 33% (1/3) on skills [118]. The measurement properties mentioned in the referenced validation studies were internal consistency and reliability, followed by internal validity.

### VR Serious Gaming and Gamification

Of the 115 studies, 12 (10.4%) with 743 medical students evaluated the effects of VR serious gaming and gamification in medical student education [125-136]. The studies included participants from the first to fifth year of studies and were published between 2008 and 2020. Regarding the topic or area of study, 25% (3/12) of the studies focused on surgery [126,129,136] and 8% (1/12) each on acute medicine [131], advanced life support [132], basic life support [127], engagement and self-efficacy beliefs [128], geriatric medicine [130], laparoscopy [135], pediatrics [133], primary care [134], and urology [125].

For the outcomes, 58% (7/12) of the studies reported on participants’ postintervention knowledge [125,127,129, 130,132-134], 58% (7/12) on skills [126,127,129, 131,132,135,136], 17% (2/12) on attitudes toward the intervention and toward the outcomes [125,132], 17% (2/12) on satisfaction [133,134], 8% (1/12) on competencies [130] and 8% (1/12) on engagement and self-efficacy belief [128](Figure 2). Of the 7 studies assessing participants’ skills, 1 (14%) also reported change score from baseline [126]. Overall, 25% (3/12) of the studies assessed retention [126,133,134]. Of these 3 studies, 2 (67%) assessing the knowledge outcome also assessed retention from 4 to 6 weeks after the intervention [133,134] and 1 (33%) assessing the skills outcome also assessed retention at 3 weeks after the intervention [126].

For the assessment methods, most of the included studies used paper-based written assessments [125,130], in-person assessments by supervising clinicians [126,131,135,136], or both assessment methods [127,129,132]. Of the 12 studies, 1 (8%) used digital assessments in the form of a questionnaire in addition to paper-based written assessment [134], 1 (8%) used only digital assessments in the form of a questionnaire [133], and the mode of assessment in 1 (8%) was not mentioned [128].

Of the 12 studies, 7 (58%) reported at least one form of validity evidence (mostly internal consistency and reliability) for the measurement instruments that were mainly used to assess knowledge [125,126,128-130,133,134] (Multimedia Appendices 2 and 3). Of these 7 measurement instruments, 4 (57%) were focused on knowledge, 2 (29%) on skills, 2 (29%) on satisfaction, and 1 (14%) each on cognitive load and self-efficacy beliefs. Of the 7 studies, 3 (43%) referenced a measurement instrument validation study [126,128,133]. The reported measurement properties included internal consistency (for the skills, engagement, and satisfaction measurement instrument), reliability (for the skills and engagement measurement instrument), structural validity (for the skills and satisfaction measurement instrument), and hypothesis (for the skills measurement instrument).

### AR Interventions

Of the 126 studies, 11 (8.7%) with 448 medical students used an AR intervention to assess the outcomes [137-147]. The studies included first year to fourth year medical students and were published between 2013 and 2020. The studies covered different topics, including arthroplasty [142], facet joint injection [143], needle insertion [147], general medicine [144], forensic medicine [137], ophthalmology [140], surgery [141,145], laparoscopy [146], and anatomy [138,139].

The reported outcomes included participants’ postintervention knowledge [137-139,144], skills [138,140-143,145-147], attitudes toward learning experience or intervention [137,140-142,144], satisfaction [138,146], emotional state , [137,144] and cognitive load [139] (Figure 2). Most studies used paper-based written assessments [137-139,144] or in-person assessments by examiners [143,147] or both approaches [140,142,146]. Of the 11 studies, 1 (9%) used both digital and paper-based written assessments [141] and 1 (9%) used digital assessment in the form of software-based metrics [145]. Of the 8 studies assessing a skills outcome, 2 (25%) also reported change score from baseline [138,145]. Similarly, of the 6 studies assessing knowledge and satisfaction, 1 (17%) also reported change score from baseline [138]. In terms of retention, only 25% (1/4) of the studies assessing knowledge also reported retention 2 weeks after the intervention [144].

Of the 11 studies, 6 (55%) reported at least one form of validity evidence (mostly internal consistency) for a variety of measurement instruments used [137-140,144,145]. These measurement instruments were used to assess knowledge in 18% (2/11) of the studies, attitudes in 18% (2/11), and emotional state in 18% (2/11), whereas in 9% (1/11) of the studies each, skills, cognitive load, and visuospatial assessment were assessed. None of the studies provided references for validation of the instruments used to measure the outcomes.

### MR Interventions

None of the included studies assessed the effectiveness of MR interventions in medical student education.

## Discussion

### Principal Findings

In this review, we assessed and mapped the choice of outcomes, measurement instruments, and the prevalence of measurement instruments with validity evidence in RCTs on the use of ER technologies in undergraduate medical education. Among the 126 included studies, we found 115 (91.3%) RCTs on different forms of VR, 11 (8.7%) articles on AR simulations, and no RCTs on MR in medical student education. The included studies often reported only a single outcome and immediate postintervention assessments. The types of reported outcomes varied across different types of VR and AR simulations. Participants’ skills were the most common outcomes measured in studies on VR simulators, VR patient simulations, and AR. Participants’ knowledge was the most common outcome measured in studies on screen-based VR and VR serious games. Other more commonly reported primary outcomes were participants’ attitudes toward the intervention or topic and satisfaction with the intervention. More than half of the studies on VR simulators, VR patient simulations, VR serious gaming, and AR as well as only a quarter of the studies on screen-based VR reported at least one form of validity evidence. The most common validity evidence for the measurement instruments used were internal consistency and reliability. Most of the studies used nondigital assessment methods such as paper-based written or in-person assessments by an examiner.

### Comparison With Existing Literature

There is a lack of standardization regarding the choice of outcomes and assessments in RCTs focusing on ER for medical student education. The findings are in line with published reviews focusing on the effectiveness of digital education for pre- and postregistration health professionals [1,131,148].

Our review shows a diversity of outcomes and measurement instruments used in trials on ER in medical education. Reporting of a limited set of outcomes, immediate postintervention data, and the use of measurement instruments lacking validity evidence is common in RCTs on different digital health professions education modalities. However, the choice of appropriate outcomes as well as robust measurement instruments to assess these outcomes is essential when designing trials. It is also important that the chosen outcomes are relevant to key stakeholders who will be able to influence policy and practice. This can be achieved through the development and use of an agreed standardized collection of outcomes and measurement instruments [21].

### Strengths and Limitations

In our review, we used a comprehensive search strategy for 7 major bibliographic databases and gray literature sources without language limitations to identify relevant studies. We covered the search period starting from 1990 onward to include all available RCTs on VR-, AR-, and MR-based trainings in medical student education. We performed the screening and data extraction in parallel and independently to ensure reliability of our findings.

There are also some limitations to our study. We performed a descriptive analysis and mapping of outcomes and validity evidence for the measurement instruments used. A more in-depth analysis of the types of validity evidence used was not feasible because of limited information in the included studies. We aimed to complement this by searching for, and including, additional information on validity evidence from validation studies referenced in the included studies. However, information provided in these referenced validation studies was also often limited. We acknowledge that some of the mentioned measurement instruments may have validity evidence not reported in the included RCT papers or for which no validity study was referenced. Furthermore, the reporting of validity evidence in the included RCTs and validation studies may be incomplete and not reflect all validity evidence for a particular measurement instrument. Finally, to determine the validity evidence for the measurement instruments used in the included trials, we used COSMIN, an established taxonomy of measurement properties. Although COSMIN was originally developed for health outcome measurement instruments, it is also applicable to other types of outcomes. However, there are other validity frameworks that were developed primarily for education and may be more appropriate for future analysis of medical education outcomes [9,149].

### Future Recommendations

Future studies should aim to include a broader set of outcomes, report change score from baseline, and assess learning retention. They should also aim to use measurement instruments with validity evidence. We list those used in the included trials in Multimedia Appendix 3. Most of the measurement instruments with validity evidence were used to assess participants’ skills. There is a need for greater use or adaptation of existing measurement instruments with validity evidence and potentially also development of new ones assessing other relevant outcomes such as attitudes and satisfaction. In addition, digital technology offers diverse and potentially more efficient approaches to assessment and should be more extensively explored and applied in this area. This is particularly relevant given the pervasive and sudden shift to remote teaching because of the COVID-19 pandemic.

### Conclusions

Studies on the use of VR and AR in undergraduate medical education often report a limited set of outcomes, mostly knowledge and skills, and usually immediate postintervention assessment data. The use of measurement instruments with validity evidence for outcomes other than skills is limited, as is the use of digital forms of assessment. Future studies should report a broader set of outcomes, change score from baseline, and retention data, as well as use measurement instruments with validity evidence.

## Appendix

Multimedia Appendix 1 MEDLINE (Ovid) search strategy.

Multimedia Appendix 2 Characteristics of the included studies.

Multimedia Appendix 3 Types and number of reported outcomes and measurement instruments with validity in the included studies.

## Abbreviations

AR: augmented reality
COSMIN: Consensus-Based Standards for the Selection of Health Measurement Instruments
ER: extended reality
MR: mixed reality
PRISMA: Preferred Reporting Items for Systematic Reviews and Meta-Analyses
RCT: randomized controlled trial
VR: virtual reality
WHO: World Health Organization
