# Supplementary material for: Outcomes, Measurement Instruments, and Their Validity Evidence in Randomized Controlled Trials on Virtual, Augmented, and Mixed Reality in Undergraduate Medical Education: Systematic Mapping Review
Source: JMIR Serious Games. 2022 Apr 13;10(2):e29594. doi: 10.2196/29594 (PMC9047880; doi:10.2196/29594)
Supplement: Multimedia Appendix 1 [file games_v10i2e29594_app1.docx]

## Virtual, augmented and mixed reality in undergraduate medical education: a systematic mapping review of the outcomes and measurement instruments in randomized controlled trials

## Multimedia Appendix

#### Multimedia Appendix 1. MEDLINE (Ovid) search strategy

1. exp education, professional/ not education, veterinary/

2. Education, Predental/

3. Education, Premedical/

4. exp Students, Health Occupations/

5. ((medic* or premedic* or dent* or laborator* or predent* or midwi?e* or nurs* or nutrition* or orthop* or podiat* or pharmac* or psycholog* or psychiatr* or health or healthcare or occupational therap* or physiotherap* or physical therap* or clinical or surg* or radiolog* or obstetric* or gyn?ecolog* or orthodont* or An?esthesi* or Dermatolog* or Oncolog* or Rheumatolog* or Neurolog* or Patholog* or P?ediatric* or Cardiolog* or Urolog*) adj3 (student* or graduate* or undergraduate* or staff or personnel or practitioner* or clerk* or fellow* or internship* or residen* or educat* or train* or novice* or tutor*)).tw,kf.

6. or/1-5

7. Computer-Assisted Instruction/

8. exp Internet/

9. Computer Simulation/

10. Patient Simulation/

11. software/

12. Mobile Applications/

13. User-Computer Interface/

14. Video Games/

15. Web Browser/

16. Education, Distance/

17. Computers/

18. exp Microcomputers/

19. exp Cell Phones/

20. Games, Experimental/

21. exp Models, Anatomic/

22. Audiovisual Aids/

23. Educational Technology/

24. Electronic Mail/

25. exp Telemedicine/

26. Telenursing/

27. Telecommunications/

28. Webcasts/

29. exp Videoconferencing/

30. ((computer* or digital* or hybrid or blended or mixed mode or distance or remote* or electronic or mobile or online* or interactiv* or multimedia or internet or web* or virtual* or game* or gaming or Videogame* or Videogaming) adj3 (classroom* or course* or educat* or instruct* or learn* or lecture* or simulat* or train* or teach* or tutor* or platform*)).tw,kf.

31. (Simulat* adj3 (course* or educat* or instruct* or learn* or train* or teach* or platform* or high-fidelity)).tw,kf.

32. e-learn*.tw,kf.

33. elearn*.tw,kf.

34. m-learn*.tw,kf.

35. mlearn*.tw,kf.

36. smartphone*.tw,kf.

37. smart-phone*.tw,kf.

38. ((mobile or cell) adj2 phone*).tw,kf.

39. iphone*.tw,kf.

40. android*.tw,kf.

41. ipad*.tw,kf.

42. Personal digital assistant*.tw,kf.

43. handheld computer*.tw,kf.

44. Mobile App?.tw,kf.

45. Mobile Application?.tw,kf.

46. webcast*.tw,kf.

47. webinar*.tw,kf.

48. flipped classroom*.tw,kf.

49. Serious game*.tw,kf.

50. Serious gaming.tw,kf.

51. Patient Simulat*.tw,kf.

52. Virtual patient*.tw,kf.

53. ((educat* or instruct* or learn* or simulat* or train* or teach* or interactiv*) adj2 technolog*).tw,kf.

54. Massive Open Online Course?.tw,kf.

55. Mooc?.tw,kf.

56. (Canvas network or Coursera or Coursesites or edx or Futurelearn or iversity or miriada x or moodle or novoed or openlearning or open2study or plato or spoc or udacity or pingpong).tw,kf.

57. or/7-56

58. 6 and 57

59. Education.fs.

60. Education/

61. Teaching/

62. Learning/

63. exp Inservice Training/

64. Curriculum/

65. educat*.tw,kf.

66. learn*.tw,kf.

67. train*.tw,kf.

68. instruct*.tw,kf.

69. teach*.tw,kf.

70. or/59-69

71. Health Personnel/

72. exp Allied Health Personnel/

73. Anatomists/

74. "Coroners and Medical Examiners"/

75. exp Dental Staff/

76. exp Dentists/

77. Health Educators/

78. Infection Control Practitioners/

79. Medical Laboratory Personnel/

80. exp Medical Staff/

81. exp Nurses/

82. exp Nursing Staff/

83. Personnel, Hospital/

84. Pharmacists/

85. exp Physicians/

86. Physician*.tw,kf.

87. Doctor*.tw,kf.

88. Nurs*.tw,kf.

89. Surg*.tw,kf.

90. Health Personnel.tw,kf.

91. healthcare professional*.tw,kf.

92. radiolog*.tw,kf.

93. dentist*.tw,kf.

94. Pharmacist*.tw,kf.

95. Hospital Administrator*.tw,kf.

96. Podiatr*.tw,kf.

97. Psycholog*.tw,kf.

98. Psychiatr*.tw,kf.

99. An?esthesi*.tw,kf.

100. Clinician*.tw,kf.

101. Dermatolog*.tw,kf.

102. General practioner*.tw,kf.

103. Cardiolog*.tw,kf.

104. Oncolog*.tw,kf.

105. Rheumatolog*.tw,kf.

106. Neurolog*.tw,kf.

107. Patholog*.tw,kf.

108. P?ediatric*.tw,kf.

109. Physiotherap*.tw,kf.

110. Physical therap*.tw,kf.

111. Occupational therap*.tw,kf.

112. dieti?ian*.tw,kf.

113. Dietetic*.tw,kf.

114. midwi?e*.tw,kf.

115. nutrition*.tw,kf.

116. orthopti*.tw,kf.

117. obstetric*.tw,kf.

118. gyn?ecolog*.tw,kf.

119. orthodont*.tw,kf.

120. Urolog*.tw,kf.

121. or/71-120

122. Health Occupations/

123. exp Allied Health Occupations/

124. Biomedical Engineering/

125. Chiropractic/

126. exp Dentistry/

127. exp Evidence-Based Practice/

128. exp Medicine/

129. exp Nursing/

130. Dietetics/

131. Optometry/

132. Orthoptics/

133. exp Pharmacology/

134. exp Pharmacy/

135. Podiatry/

136. Psychology, Medical/

137. Serology/

138. Specialization/

139. exp Surgical Procedures, Operative/

140. exp Radiography/

141. or/122-140

142. 121 or 141

143. 57 and 70 and 142

144. Psychomotor Performance/

145. motor skills/

146. ((psychomotor or procedural or technical) adj3 skill*).tw,kf.

147. (psychomotor adj3 performance).tw,kf.

148. or/144-147

149. 6 and 148

150. 58 or 143 or 149

151. limit 150 to yr="1990 -Current"

152. randomized controlled trial.pt.

153. controlled clinical trial.pt.

154. randomized.ti,ab.

155. placebo.ti,ab.

156. drug therapy.fs.

157. randomly.ti,ab.

158. trial.ti,ab.

159. groups.ti,ab.
