# Supplementary material for: Outcomes, Measurement Instruments, and Their Validity Evidence in Randomized Controlled Trials on Virtual, Augmented, and Mixed Reality in Undergraduate Medical Education: Systematic Mapping Review
Source: JMIR Serious Games. 2022 Apr 13;10(2):e29594. doi: 10.2196/29594 (PMC9047880; doi:10.2196/29594)
Supplement: Multimedia Appendix 2 [file games_v10i2e29594_app2.docx]

#### Multimedia Appendix 2. Characteristics of the included studies

1. Studies on virtual reality simulators

| **1.Study ID, Country, Number of medical students (n), Study year** | **Name of instrument (VR-simulator)** | **Type of comparison(s)** | **Subjects/topics** | **Assessment mode (online/offline/**  **hardcopy)** | **Knowledge** | **Skills** | **Attitude** | **Satisfaction** | **Others** | **Validation** |
| --- | --- | --- | --- | --- | --- | --- | --- | --- | --- | --- |
| Aggarwal 2006, Denmark, n=20, unspecified | MIST-VR | VR vs VR | Laparoscopy | Offline Computer-based checklists | . | Time, economy of movement, total error | . | . |  | Yes |
| Ahad 2013, USA, n=32, third and fourth year | AccTouch colonoscopy simulator (Immersion Medical, MD) | VR vs traditional learning | Colonoscopy | Offline Computer-based assessments | . | Checklists (time, scope-path length, percentage of air left) | . | . |  | Yes (9 out of 14 parameters were previously validated) |
| Ahlberg 2002, Sweden, n=29, fourth year | MIST-VR | VR vs traditional learning (no training) | Laparoscopy | Assessments by supervision clinicians | . | Hardcopy checklists (the operations were videotaped and examined by three independent observers.) | . | . |  | . |
| Anderson 2015, Denmark, n=43, unspecified | The Visible Ear Simulator | VR vs VR | Surgical skills training | Assessments by supervision clinicians | . | Scale (26-items) | . | . | Mean reaction time (for cognitive load) | Yes |
| Bergqvist 2014, Denmark, n=20, unspecified | Eyesi cataract simulator | VR vs VR | Ophthalmology training | Offline Computer-based checklists | . | Checklists (Time, checklists) | . | . | . | . |
| Bjerrum2016, Denmark, n=96, unspecified | LapSim (virtual reality laparoscopy simulators) | VR vs VR | Laparoscopy | Both Offline Computer-based assessment and hard copy checklists | . | Checklists (Time, number of repetitions) | . | . | Subjective Mental Effort Questionnaires (SMEQ: 0-150) (for assessing cognitive load) | Yes (for cognitive load) |
| Bowyer 2005 (teaching intravenous cannulation), USA, n=34, third year | Virtual IV and CathSim | VR1 vs traditional learning, VR2 vs traditional learning | Intravenous cannulation | Offline Computer-based assessment | . | Checklists | . | . | . | . |
| Bowyer 2005 (validation) USA, n=40, third year | SimPL | VR vs traditional learning | Surgical skills training | Unspecified | Questionnaires (30 items test) | Checklists, rating scales (Likert scales) | . | . | . | . |
| Brunckhorst 2015, UK, n=32, unspecified | URO Mentor | VR vs traditional learning | Ureteroscopy | Unspecified | . | Time to completion, OSATS, RUES, NOTSS | . | . | . | Yes |
| Bruwaene 2015, Belgium, n=30, unspecified | Lap Mentor | VR vs traditional learning | Laparoscopy | Assessments by supervision clinicians | . | Time, checklists, validated rating scale (5 global and 3 specific rating items: 5-point rating scale) | Questionnaires (5-point Likert scale) | . | . | Yes (for skills outcome) |
| Brydges 2010, Canada, n=45, unspecified | Virtual IV | VR vs traditional learning | Intravenous cannulation | Assessments by supervision clinicians | . | IPPI rating tool (7-point rating scale), validated GRS and Checklist (OSATS), Ratings from experts | Questionnaires (5-point Likert scale) | . | . | Yes |
| Bube 2020, Denmark, n=32, final years (last two years) | URO Mentor | VR vs traditional learning | Ureteroscopy | Assessments by supervision clinicians | . | Global Rating Scale (5-point Likert scale) | . | . | . | Yes |
| Chou 2006, USA, n=16, first year | Bimbionix UROmentor VRS | VR vs other form of DE (offline computer-based training) | Ureteroscopy | Assessments by supervision clinicians | . | OSATS | . | . | . | Yes |
| da Cruz 2010, Brazil, n=15, first to fourth year | Lap VR | VR (more simulations) vs traditional learning (no training), VR (less simulations) vs traditional learning (no training) | Laparoscopy | Assessments by supervision clinicians | . | Checklists (time, checklist on volume of blood lost) | . | . | . | Yes |
| da Cruz 2016, Brazil, n=20, fifth and sixth year | Lap VR | VR vs traditional learning | Laparoscopy | Assessments by supervision clinicians | . | Checklists (time, checklist on volume of blood lost) | . | . | . | Yes |
| De La Garza 2019, n=80, third to sixth year | Lap Mentor | VR vs VR | Laparoscopy | Both Hard copy and in person assessments with checklist | MCQ | OSAT | . | . | . | Yes |
| Eldred-Evans 2013, UK, n=64, unspecified | Lap VR | Blended VR vs traditional learning | Laparoscopy | Both offline computer- based metric and hard copy checklists | . | Checklists (time, checklists on accuracy, precision, and overall performance) | . | . | . | . |
| Frithioff 2020, Denmark, n=24, Unspecified | Visible Ear Simulator | VR vs VR | Orthopedic surgery | Assessments by supervising clinicians | . | Modified Welling scale | . | . | Reaction time (for measuring cognitive load) | Yes |
| Fu 2019, USA, n=14, first to third year | VBLaST-SS | VR vs traditional learning | Laparoscopy | Offline Computer based assessment matrix (Cumulative summation) | . | Clinical simulation | . | . | . | Yes |
| Ganai 2007, USA, n=20, third year | Telescope simulator (EndoTower software) | VR vs traditional learning (no training) | Laparoscopy | Computer based assessments (real time) | . | Checklists (time, scope path length, scope rotation path length and % of time) | . | . | . | . |
| Gasco 2014, USA, n=26, unspecified | ImmersiveTouch Simulator | VR vs traditional learning (no training) | Orthopedic surgical skills training | Computer based error analysis (number of errors made) | . | Checklists (number of errors made) | . | . | . | . |
| Getterman 2019, USA, n=421, second year | Mpathic-VR | VR vs offline computer-based learning | Empathic communication skills | Assessments by supervising clinicians |  | In person assessment using checklists and scale | . | . | . | . |
| Hariri 2004, USA, n=29, first year | Procedicus (Virtual Reality Arthroscopy simulator) | VR vs traditional learning (textbook graphics) | Learning shoulder joint clinical anatomy | Both Offline Computer-based checklists and hard copy assessments | Test questionnaires/  images (7 images) | . | Questionnaires (5-point Likert scale) | . | . | . |
| Hiemstra 2011, Netherland, n=50, unspecified | SIMENDO VR trainer | VR vs traditional learning | Laparoscopy | Offline Computer-based checklists | . | Checklists (time, total path length, motion in depth) | . | . | . | . |
| Hyltander 2002, Sweden, n=24, unspecified | LapSim (virtual reality laparoscopy simulators) | VR vs traditional learning (no training) | Laparoscopy | Assessments by supervising clinicians | . | Rating scale (9-steps) | . | . | . | . |
| Johnston 2013, UK, n=20, unspecified | iSIM (integrated Laproscopic Simulator) | VR vs traditional learning (no training) | Laparoscopy | Assessments by supervising clinicians | . | OSATS (validated) | . | . | . | Yes |
| Kanumuri 2008, USA, n=16, third year | MIST-VR | VR vs other form of DE (offline computer-based training) | Laparoscopy | Both Computer-based metrics (offline) and hard copy questionnaires | . | Checklists (time, path length and smoothness of motion) | Surveys | . | . | Yes (for skills outcome) |
| Karabanov 2019, Denmark, n=48, unspecified | LapSim | VR vs traditional education | Endoscopy | Offline Computer-based metrics (based moment speed and time) | . | Clinical simulation | . | . | . | . |
| Kothari 2002, USA, n=29, third year | MIST-VR | VR vs other form of DE (offline computer-based training) | Laparoscopy | Offline Computer-based assessment | . | Time to completion | . | . | . | . |
| Kowalewski 2019, Germany, n=100, third to six year | . | VR vs VR vs None | Laparoscopy | Assessments by supervising clinicians | . | OSAT | . | . | . | Yes |
| Krogh 2013, Denmark, n=20, third to sixth year | Orsim brochoscopy simulator | VR vs traditional learning (no manual training) | Laparoscopy | Assessments by supervising clinicians | . | Validated bronchoscopy quality test | . | . | . | Yes |
| Lee 2019, South Korea, n=64, | da Vinci Surgical system | VR vs non-VR surgical skills simulator | Surgical skills | In person (checklists and assessed with blind raters) and written assessments | . | Multisource (360-degree) assessments [In person (checklists and assessed with blind raters) plus self-assessment questionnaire] | . | . | . | Yes |
| Lesch 2020, USA, n=37, second, third and fourth year | TIPS (Toolkit for Illustration of Procedures in Surgery) | VR vs Online digital education | Laparoscopy | Hardcopy written exercises | MCQs | . | Surveys | . | . | Yes |
| Lindquist 2019, USA, n=34, | Endoscopic Sinus Surgery Simulator | VR vs traditional education | Surgical skills | Assessments by supervising clinicians |  | Global rating scale | . | . | . | Yes |
| Loukas 2012, Greece, n=44, unspecified | Lap VR | VR vs other form of DE (offline computer-based training) | Laparoscopy | Computer based assessment metrics | . | Checklists (Time, number of errors (penalty score), instrument path length) | . | . | . | . |
| Loukas 2015, Greece, n=36, unspecified | Lap VR | VR vs VR | Laparoscopy | Computer based assessment metrics | . | Checklists (Time, number of errors and instrument path length) | . | . | . | . |
| Lucas 2008_VR training improves, USA, n=32, first and second year | Lap VR | VR vs traditional learning (no training) | Laparoscopy | Assessments by supervising clinicians | . | OSATS | . | . | . | Yes |
| Madan 2007_Propsective random, USA, n=65, first and second year | MIST-VR | VR vs traditional learning (box trainer or no training), Blended VR vs traditional learning (box trainer or no training) | Laparoscopy | Offline Computer-based assessments | . | Checklists (time and number of errors) | . | . | . | . |
| McDougall 2009, USA, n=20, unspecified | Simbionix LAP Mentor | VR vs traditional learning (pelvic box trainers) | Laparoscopy | Assessments by supervising clinicians | . | OSATS | . | . | . | Yes |
| Mulla 2012, USA, n=41, unspecified | Lap VR | VR vs traditional learning (no training); VR vs traditional learning (box trainers); VR vs traditional learning (box trainers plus additional training) | Laparoscopy | Offline Computer-based assessment | . | Checklists (time, scales on Precision, Accuracy, Performance) | . | . | . | . |
| Munz 2004, UK, n=24, unspecified | LapSim (virtual reality laparoscopy simulators) | VR vs other form of DE (offline computer-based training), VR vs traditional learning | Laparoscopy | Offline Computer-based assessment | . | ICSAD (motion tracker) | . | . | . | . |
| Muresan 2010, USA, n=41, first to fourth year | Lap VR | VR vs traditional learning | Laparoscopy | Both online computer- based online assessment and hard copy assessment | . | Checklists (Time, knot quality, suture placement accuracy, and mental workload) | . | . | Cognitive load | Yes (for cognitive load) |
| Nehme 2013, UK, n=30, third year | ENDO-TS-1, MIST-VR | VR vs VR, VR vs traditional learning (no training) | Laparoscopy | Offline computer-based assessment metrics | . | Checklists (Time, number of errors) | . | . | . | Yes |
| Neumann 2019, Germany, n=51, fourth year | Uro-Trainer | VR vs offline computer-based training | Ureteroscopy | Offline computer-based metrics | . | Clinical simulations | . | . | . | . |
| Nickel 2015, Germany, n=84, unspecified | LAP Mentor II | VR vs other form of DE (blended online training) | Laparoscopy | Offline Computer-based assessments | Questionnaires (16 items) | OSATS | Checklists (5-point Likert scale) | . | . | Yes (for skills outcome) |
| Oussi 2020, Sweden, n=63, fourth year | Lap Mentor | VR vs traditional | Laparoscopy | Assessments by supervising clinicians | . | Clinical simulations | Questionnaires | . | . | Yes |
| Patel 2014, USA, n=20, unspecified | ImmersiveTouch Simulator | VR vs traditional learning (no training) | Surgical skills training | Unspecified | . | Number of detection (objects: small to large) | . | . | . | . |
| Plana 2019, USA, n=35, first year | . | VR vs traditional | Surgical simulation | Assessments by supervising clinicians and hard copy written exercises | . | Checklists | . | Surveys (Validated Educational Quality Survey) | . | Yes |
| Schlosser 2007, Germany, n=14, unspecified | LapSim (virtual reality laparoscopy simulators) | VR vs VR | Laparoscopy | Offline Computer-based assessment metrics | . | Checklists (time, checklists) | . | . | . | . |
| Selvander 2012, Sweden, n=35, unspecified | Eyesi cataract simulator | VR vs VR | Ophthalmology training | Assessments by supervising clinicians | . | Checklists | . | . | . | Yes |
| Solyar 2008, USA, n=15, first year | The Endoscopic Sinus Surgery Simulator (ES3) | VR vs traditional learning | Surgical skills training | Assessments by supervising clinicians and hard copy written exercises | . | Checklist questionnaires | Survey | Survey | . | . |
| Sotto 2009, Philippines, n=40, third and fourth year | Virtual IV | VR vs traditional learning (in person practicing) | Intravenous cannulation | Assessments by supervising clinicians | . | Checklists | . | . | . | Yes |
| Sugand 2015, UK, n=52, unspecified | Trauma Vision VR | VR vs VR | Orthopedic surgical skills training | Offline Computer based assessment metrics | . | Checklists | . | . | . | . |
| Suh 2010, USA, n=14, unspecified | dVSS (da Vinci Surgical System) stimulator | VR vs other form of DE (3D game) | Surgical skills training | Unspecified | . | Time to completion | . | . | . | . |
| Sun 2014, China, n=480, unspecified | Eyesi cataract simulator | Blended VR vs traditional learning | Ophthalmology training | Assessments by supervising clinicians and hard copy written exercises | . | Checklists (through case analysis by three examiners) | Questionnaires | . | . | . |
| Tanoue 2005, Japan, n=30, unspecified | Procedicus (Vritual Reality Arthroscopy simulator) | VR vs other form of De (offline computer-based training) | Surgical skills training | Unspecified | . | Checklists (time to completion, number of error) | . | . | . | . |
| Tanoue 2008, Japan, n=55, unspecified | Procedicus (Vritual Reality Arthroscopy simulator) | VR vs traditional learning (box trainer), VR vs traditional learning (control) | Surgical skills training | Unspecified | . | Checklists (time to completion, number of error) | . | . | . | . |
| Torkington, UK, n=30, unspecified | MIST-VR | VR vs traditional learning (conventional, VR vs traditional learning (no training) | Laparoscopy | Computer based assessment metrics | . | Checklists (tracking device (validated) | . | . | . | Yes |
| Unger 2016, Canada, n=20, second year | GeoMagic Touch | VR vs VR | Orthopedic surgical skills training | Hardcopy checklists | . | Scale | . | . | . | Yes |
| Wilhelm 2002, USA, n=21, unspecified | Simbionix Uromentor trainer | VR vs traditional learning (no further training) | Ureteroscopy | Assessment by supervising clinicians and evaluations | . | Multisource (360-degree) assessments [Checklists - time, global rating scale, self-evaluation, performance checklists] | . | . | . | Yes (for global rating scale) |
| Youngblood 2005, USA, n=46, first and second year | LapSim (virtual reality laparoscopy simulators) | VR vs traditional learning (no trainer), VR vs traditional learning (Tower Trainer system) | Laparoscopy | Assessments by supervising clinicians | . | Checklists (time, accuracy scores, global rating scores) | . | . | . | . |
| Zeltser 2007, USA, n=32, first and second year | Lap Mentor | VR vs traditional learning (no training) | Laparoscopy | Assessments by supervising clinicians | . | OSATS | . | . | . | Yes |
| Zhang 2013, USA, n=18, unspecified | VBLaST-PT | VR vs traditional learning (box trainer), VR vs traditional learning (no training) | Laparoscopy | Unspecified | . | Checklists (time and number of errors made) | . | . | . | . |
| Zhao 2011_can virtual reality, Australia, n=20, unspecified | Mediseus Surgical drilling simulator | VR vs traditional learning | Orthopedic surgical skills training | Assessments by supervising clinicians | . | OSATS, Welling Scales | . | . | . | Yes |

1. Studies on screen-based virtual reality

| **Study ID, Country, Number of medical students, Year of study** | **Type of comparison(s)** | **Type of intervention (3D, VR, Level of interactivity)** | **Assessment mode (online/offline/hardcopy)** | **Knowledge** | **Skills** | **Attitude** | **Satisfaction** | **Others** | **Validation** |
| --- | --- | --- | --- | --- | --- | --- | --- | --- | --- |
| Alverson 2008, USA, n=36, second year | VR vs traditional learning | VR environment | Hardcopy-based assessment | . | . | Rating Scale (5-point) | . | . | . |
| Battulga 2012, Japan, n=100, first year | VR vs traditional learning | 3D | Hardcopy-based assessment | . | . | Rating Scale (5-point Likert scale) | Rating Scale (5-point Likert scale) | . | . |
| Blumstein 2020, USA, n=20, first and second year | VR vs traditional learning | 3D | Assessment by supervising clinicians | . | Global Assessment 5-Point Rating Scale and Procedure Specific Checklist | . | . | . | Yes |
| Courteille 2013, Sweden, n=41, fourth year | VR vs other form of DE (offline computer-based learning) | VP/VHP within VR | Hardcopy-based assessment | MCQ (12 items) | . | . | Questionnaires (9 items) | Students' learning engagement (questionnaires: 9 items). | . |
| Deladisma 2009, USA, n=29, third year | VR vs traditional learning | VP/VHP within VR | Hardcopy-based assessment | . | . | Rating Scale  (5-point) | . |  | . |
| Drapkin 2015, USA, n=62, first year | VR vs VR | 3D | Hardcopy-based assessment | Questionnaires (32 items) | . | Rating Scale  (5-point Likert scale) | Rating Scale (5-point Likert scale) | . | Yes |
| Flores 2013, USA, n=32, first and second year | VR vs traditional learning | 3D animation | Hardcopy-based assessment and in person assessments | . | Checklists (time, Arch Bar Placement Assessment Scale (validated)) | . | Student Evaluation of Educational Quality Survey (validated) | . | Yes |
| Glittenberg 2003, Austria, n=42, unspecified | VR vs traditional learning | 3D | Hardcopy-based assessment | MCQ | . | . | Survey (questionnaires) | . | . |
| Glittenberg 2006, Austria, n=100, unspecified | VR vs traditional learning | 3D | Hardcopy-based assessment | MCQ (26 items) | . | Survey  (12 items) | . | . | . |
| Gutierrez 2007, USA, n=25, first year | VR vs VR | VR environment (fully immersive) | Offline Computer based scaling algorithm (Pathfinder) | Ratings | . | . | . |  | . |
| Hampton 2010, USA, n=43, third and fourth year | VR vs traditional learning | 3D | Hardcopy-based assessment | 21 questionnaires | . | Rating Scale  (5-point Likert scale) | . |  | . |
| Hisley 2008, USA, n=16, first year | VR vs traditional learning | 3D | Combined assessment (computer- and paper-based assessment (questions via PPT slides and answers with a piece of paper) | Questionnaires | . | . | . | . | . |
| Hopkins 2011, Canada, n=74, unspecified | VR vs traditional learning, Blended VR vs traditional learning | 3D | Hardcopy-based assessment | MCQ (14 items) | . | Survey (questionnaires) | Survey (questionnaires) | . | . |
| Hu 2016, USA, n=100, unspecified | VR vs traditional learning | 3D models | Hardcopy-based assessment | MCQ | . | . | Rating Scale (5-point Likert scale) | . | Yes |
| Kalet 2012, USA, n=143, second year | VR vs VR | 3D models | Hardcopy-based assessment | MCQ (18 items) | Checklists (17 items) | . | . | . | Yes |
| Keedy 2011, USA, n=46, first and fourth year | VR vs other form of DE (online/computer-based learning) | 3D models | Hardcopy-based assessment | MCQ | . | . | Rating Scale (5-point Likert scale) | . | . |
| Khatib 2014, UK, n=56, unspecified | VR vs traditional learning | VRE surgical stimulation | Hardcopy-based assessment and in person assessments | MCQ (20 items) | Objective rating scale | . | . | . | Yes (for skills: content and face validity) |
| Kockro 2015, Germany, n=169, second year | VR vs other form of DE (online/computer-based learning) | 3D models | Hardcopy-based assessment (written exercises) | MCQ (10 items) | . | Survey (questionnaires) | Survey (questionnaires) . | . | . |
| Lorenzo-Alvarez 2019, Spain, n=215, third year | VR vs traditional learning | 3D models | Hardcopy-based assessment (written exercises) | Questionnaires (12 items) | . | . | . | . | . |
| Maresky 2019, Canada, n=42, first year | VR vs traditional learning | VR Environment | Hardcopy-based assessment (written exercises) | MCQ (10 items) | . | Questionnaires | . | . | . |
| Motsumi 2019, Africa, n=90, third and fifth year | VR vs traditional learning | VR environment | Online-based assessment on Moodle | Test questionnaires | . | . | Survey (questionnaires) | . | . |
| Nicholson 2006, Canada, n=61, first year | VR vs other form of DE (online/computer-based learning) | 3D models | Web-based assessment | MCQ (12 items) | . | . | Survey (questionnaires) | . | . |
| Patel 2012, UK, n=60, first year | VR vs traditional learning | VP/VHP within VR | Hardcopy-based assessment | MCQ | Observation scale (Likert-type) | Survey (Likert-type checklist) | . | . | Yes |
| Persky et al_medical student bias, USA, n=76, third and fourth year | VR vs VR | VP/VHP obese | Hardcopy-based assessment | . | . | Surveys (7-point Likert scale) | . | . | Yes |
| Prinz 2005, Austria, n=172, unspecified | VR vs other form of DE (online/computer-based learning) | 3D models | Hardcopy-based assessment | MCQ (19 items) | . | . | Survey (4 level-questionnaires) | . | . |
| Schutte 1997, Netherlands, n=68, first year | Blended VR vs traditional learning | 3D models + textbooks | Hardcopy-based assessment | Questionnaires (28 closed and 5 open questionnaires) | . | . | . | . | Yes |
| Scoville 2007, USA, n=96, first year | VR vs traditional learning | VR environment | Hardcopy-based assessment | Questionnaires (20 items) | . | . | . | . | . |
| Succar 2010, Australia, n=147, unspecified | VR vs traditional learning | VR environment | Hardcopy-based assessment | Questionnaires (20 items) | . | . | Questionnaires (20 items) | . | . |
| Succar 2013, Australia, n=188, unspecified | VR vs traditional learning | VP/VHP within VR | Hardcopy-based assessment | MCQ (20 items) | . | . | Questionnaires (9 items-5 point Likert scale) | . | . |
| Yi 2009, China, n=60, second year | VR vs traditional learning, VR vs offline digital learning | 3D models | Hardcopy-based written exercise | Post-test  (24 items) | . | . | . | Questionnaires (4 evaluation questions, 5-point Likert scale) | . |

1. Studies on virtual reality patient simulations

| **1. Study ID** | **Type of comparison** | **Types of VP** | **Subject** | **Assessment mode (online/offline/hardcopy)** | **Knowledge** | **Skills** | **Attitude** | **Satisfaction** | **Patient-related outcomes/others** | **Validation** |
| --- | --- | --- | --- | --- | --- | --- | --- | --- | --- | --- |
| Botezatu 2010_VPS for learning assessment: Superior results, Sweden, n=216, fourth year | VP vs traditional learning, Blended VP vs traditional learning | Web-based VP | Internal medicine | Combined assessments (Web-based VP plus paper-based exam) | . | Scoring rubric (0-6) | . | . | . | Yes |
| Deladisma 2007, USA, n=84, second year | VP vs traditional learning | VP on projector screen (computer-based) | Communication skills | Hardcopy-based assessment | . | Likert-type scale | . | . | Likert-type scale (for emphatic behavior) | Yes |
| Dickerson 2006, USA, n=17, second and third year | VP vs VP | VP on projector screen (computer-based) | Communication skills | Hardcopy-based assessment | . | Questionnaires and Expert evaluation | . | . | . | Yes |
| Foster 2015, USA, n=67, second year | VP vs online | Web-based VP | Suicide risk assessment | Computer -based (Both online and offline computer-based assessments) | . | Checklist (14 items) | . | Survey (16 items-online survey) | Rapport scores/checklist (5 items: 5-point Likert scales) MSIPQ | Yes (for skills and patient's satisfaction) |
| Foster 2016, USA, n=70, first year | 1) VP vs VP, 2) VP vs VP | Web-based VP | Communication skills | Computer -based (Both online and offline computer-based assessments) | . | Checklist questionnaires | . | Survey (16 items-online survey) | Rapport scores/checklist (5 items: 5-point Likert scales) MSIPQ | Yes (for skills and patient's satisfaction) |
| Lehmann 2015, Germany, n=57, third and fourth year | Blended VP vs traditional learning | Web-based VP | Basic Life Support Procedure | Hardcopy-based assessment | Questionnaires (21 items) and self-assessment items (7 items) | Checklists and self-assessment items (7 items) | . | . | . | . |
| Lehmann 2019, Germany, n=103, fifth year | VP vs offline digital education | Web-based VP | Pediatric life support skills | Assessments by supervising clinicians | . | Checklists (overall competency, temporal demands, adherence to guideline-based algorithm, quality of procedural steps) | . | . | . | . |
| McCoy 2015, USA, n=108, first year | VP vs traditional learning | Web-based VP | Clinical skills | Combined assessment (hardcopy- and computer-based assessment)  Survey (for satisfaction, electric version) | . | Diagnostic competency tasks/checklists | . | Survey (28-items) | Sub-item checklist-(for engagement) | . |
| O’Rourke 2020, n=60, USA, third year and above | VP vs standardized patient (traditional learning) |  |  | Both written and assessments by supervising clinicians | . | Scale (The Standardized Patient Checklist and Rating Scale (SPCRS), 17-item measure) | Scale (Maastricht Assessment of the Simulated Patient) | Scale | . | . |

1. Studies on virtual reality serious games

| **1.Study ID** | **Type of comparison** | **Type of intervention (3D, VR, Level of interactivity)** | **Revised Subject** | **Assessment mode (online/offline/hardcopy)** | **Knowledge** | **Skills** | **Attitude** | **Satisfaction** | **Others** | **Validation** |
| --- | --- | --- | --- | --- | --- | --- | --- | --- | --- | --- |
| Boeker 2013, Germany, n=145, third year | SGG vs Traditional learning | Offline computer based SGG (Uro-island) | Urology | Hardcopy written exercises | 34 single choice items (True/False item), validated | . | 3 items checklist (4-point Likert scale) | . | . | Yes (for knowledge outcome) |
| Boyle 2011, Ireland, n=22, third year | SGG vs Digital Psychomotor Skills trainer | Nintendo Wii | Laparoscopy | In person assessments | . | Time to complete the task, number of beads dropped, instrument path length (IPL), instrument smoothness (IS) and number of perforations | . | . | . | . |
| Chien 2013, USA, n=14, unspecified | SGG vs DPST | 3D game | Surgery | In person assessments | . | Times to completion: 1) bimanual carrying 2) ped transfer | . | . | . | . |
| de Araujo 2016, Brazil, n=20, first year | SGG vs SGG, SGG vs Traditional | Nintendo Wii [3D game (Surgi), 3D game (Shorter), 3D (racing)] | Surgery | In person assessments | . | OSATS | . | . | . | Yes |
| De Sena 2019, Brazil, n=45, first year | SGG vs traditional learning | 3D learning environment | Basic Life Support | Both hardcopy written and in person assessment | MCQ (10 questions) | Assessments by supervising clinicians | . | . | . | . |
| Hedman 2013, Sweden, n=40, second, third and fourth year | SGG vs SGG, SGG vs Traditional | Half-life (Sierra online) PC game chessmaster 10 edition, Ubisoft (PC games) | Engagement modes and Self-efficacy beliefs | Unspecified | . | . | . | . | Validated subscale (7 point Likert-type) (for engagement and self-efficacy beliefs) | Yes (for Self-efficacy beliefs) |
| Kolga Schlickum 2008, sweden, n=22, third and fourth year | SGG vs SGG | First person shooter (FPS) game (3D game) | Surgery | Both hardcopy written and in person assessment | WAIS-III NI Block repetition test | MRT-A test | . | . | . | Yes |
| Lagro 2014, Netherland, n=134, fifth year | SGG vs Traditional learning | GeriatriX (online game) | Geriatric Medicine | Hardcopy written assessment | Questionnaires, validated- 18 items | . | . | . | Questionnaires (for competency) | Yes |
| Middeke 2020, Germany, n=69, Fifth year | SGG vs SGG | SGG in 3D learning environment | Acute Medicine | In person assessments | . | Checklists | . | . | . | . |
| Phungoen 2020, Thailand, n=105, Fifth year | SGG vs Traditional learning | SGG in mobile 3D learning environment | Advanced life support | Both hardcopy written exercises and in person assessment | MCQ (50 items) | Checklists | Questionnaires | Questionnaires (9 point) | . | . |
| Sward 2008, USA, n=100, third year | SGG vs Online learning (self-study via online links) | Web-based, interactive modification of the Pediatric Board Game | Pediatrics | Online assessment | 100 items MCQs | . | . | Questionnaires (9 point) | . | Yes (for satisfaction) |
| Tubelo 2019, Brazil, n=27, second year |  |  | Primary care screening | Both hardcopy (paper-based) and online computer-based assessment | Questionnaires (10 questions) | . | . | Survey (5 points) | . | Yes |

1. Studies on augmented reality

| **Study ID** | **Type of comparison** | **Types of AR** | **Subject** | **Assessment mode (online/offline/hardcopy)** | **Knowledge** | **Skills** | **Attitude** | **Satisfaction** | **Competency** | **Others** | **Validation** |
| --- | --- | --- | --- | --- | --- | --- | --- | --- | --- | --- | --- |
| Albrecht 2013, Germany, n=10, third year | Mobile Augmented reality vs traditional | iPhone 4 with mARBble Forensics | Forensic medicine | Offline-hardcopy | Questionnaires (10 item single choice) | . | AttrakDiff2 Questionnaires (28 items - 7-point Likert scale) | . | . | Profile of Mood states questionnaire | Yes |
| Bogomolova 2019, Netherland, N=63, first and second year | AR vs traditional learning (2D atlas) | Stereoscopic AR | Anatomy | Hardcopy written exercises | Questionnaires (30 item knowledge test) | . | . | Questionnaire 5-point Likert scale | . | . | Yes |
| Kucuk 2016, Turkey, n=80, second year | Mobile Augmented reality vs traditional | Aurasma platforms with software | Anatomy | Offline-hardcopy | Questionnaires (30MCQ questions) | . | . | . | . | Scales (self-reported Cognitive load scale: 9 point) | Yes |
| Leitritz 2014, Germany, n=37, fourth year | AR vs traditional (conventional training with graphs and pictures) | Eyesi indirect system | Ophthalmoscopy | Offline-hardcopy | . | Rating scales (validated with content expert) | Questionnaires (surveys) | . | . | . | Yes |
| Lemke 2020, Canada, n=44, second year | AR vs traditional learning | Microsoft HoloLens platform | Surgery | Both online computer-based and hard copy written assessments | . | Clinical simulations | Survey questionnaires (seven-point Likert scale,) | . | . | . | . |
| Logishetty 2019, UK, n=24, unspecified | AR vs traditional learning | AR headset | Arthroplasty | Both hardcopy written and in person assessment | . | Checklists (Scale of accuracy measure - orientation error) | Questionnaires (surveys: 10-point Likert scales.) | . | . | . | Yes (for attitude outcome) |
| Moult 2013, Canada, n=26, unspecified | Perk Tutor vs US machine | Perk Tutor Phantom (3D slicer) | Facet joint injections | Offline - computer | . | Checklists (time, distance) assessed by physicians | . | . | . | . | . |
| Noll 2017, Germany, n=44, third year | mAR vs m-learning | iPhone mARble-dermatology | General medicine | Offline and online | Questionnaires (10 item single choice) | . | AttrakDiff2 Questionnaires (28 items - 7-point Likert scale) | . | . | Profile of Mood states questionnaires (for emotional state outcome) | Yes (for emotional state outcome) |
| Sugand 2019, UK, n=45 | AR vs AR | AR (fluoroscopic simulator) | Surgery | Offline - computer-based metrics | . | Clinical simulations | . | . | . | . | Yes |
| Vera 2014, USA, n=19, unspecified | Augment telemonitoring vs traditional mentoring | AR telemonitoring platform | Laparoscopy | Offline - computer-based assessments | . | Checklists (time and number of mistakes) | . | Survey questionnaires | . | . | . |
| Yeo 2018, Canada, n=56, unspecified | Perk Tutor vs traditional | 3D slicer (www.slicer.org) | Needle insertions | Offline - computer-based recording | . | Checklists (time and number of mistakes) | . | . | . | . | . |
