# Supplementary material for: Outcomes, Measurement Instruments, and Their Validity Evidence in Randomized Controlled Trials on Virtual, Augmented, and Mixed Reality in Undergraduate Medical Education: Systematic Mapping Review
Source: JMIR Serious Games. 2022 Apr 13;10(2):e29594. doi: 10.2196/29594 (PMC9047880; doi:10.2196/29594)
Supplement: Multimedia Appendix 3 [file games_v10i2e29594_app3.docx]

#### Multimedia appendix 3. Measurement instruments with validity evidence in the included studies

| **Types of VR- and/or AR based trainings** | **Type and number of reported outcomes and measurement instruments with validity evidence** | **Measurement instruments with validity evidence used in the studies** |
| --- | --- | --- |
| VR-simulators | - Skills (n=62); 36 of 64 used instruments with validity evidence - Knowledge (n=5) - Attitude (n=9) - Satisfaction (n=2), 1 measurement instrument with validity evidence (110) - Cognitive Load (n=4) | ***Skills***: OSATSS, OSACSS, Modified Welling Scale  ***Satisfaction***: Validated Educational Quality Survey |
| Screen-based VR | - Knowledge (n=24); 3 of 24 studies used instruments with validity evidence - Satisfaction (n=12); 2 of 12 studies used instruments with validity evidence - Attitude (n=12); 2 of 12 studies used instruments with validity evidence - Skills (n=5), 4 out of 5 studies used instrumented were validated - Learning Engagement (n=1) | ***Knowledge***: Multiple Choice Questionnaires,  ***Skills***: Objective rating scale,  Arch Bar Placement Assessment Scale  ***Attitude***: Survey (Likert-type checklist)  ***Satisfaction***: Student Evaluation of Educational Quality Survey |
| VR patient simulations | - Skills (n=9), 5 of 9 studies used measurement instruments with validity evidence - Knowledge (n=1) - Satisfaction (n=3) - Patient outcomes (n=2), both instruments had validity evidence (67, 68) - Attitude (n=1) - Engagement (n=1) - Empathy (n=1) | ***Skills***: Maastricht Assessment of the Simulated Patient  ***Patient outcome:*** Standardized patient communication checklist and the Medical Student Interview Performance Questionnaire (MSIPQ). |
| VR serious gaming and gamification | - Knowledge (n=7), 2 of 7 studies used measurement instruments with validity evidence - Skills (n=7), 2 of 7 studies used measurement instruments with validity evidence - Attitude (n=2) - Satisfaction (n=2), both used measurement instruments with validity evidence - Competency (n=1), - Engagement and Self Efficacy Beliefs (n=1), used a validated assessment tool to measure the outcome | ***Knowledge***: validated questionnaires  ***Skills***: OSATS,  Validated subscale (7 point Likert-type) |
| AR | - Skills (n=7); 1 of 7 studies used measurement instruments with validity evidence - Knowledge (n=4), 2 of 4 used measurement instruments with validity evidence - Attitude (n=5), 2 of 5 used measurement instruments with validity evidence - Emotional state (n=2), both studies used measurement instruments with validity evidence - Satisfaction (n=2) - Cognitive Load (n=1), used validated instrument | ***Emotional state:*** Profile of Mood states questionnaire  ***Skills***: Rating scales (validated with content expert)  ***Attitude:*** AttrakDiff2 Questionnaires |

OSACSS - Objective Structured Assessment of Cataract Surgical Skill, OSATSS - Objective structured assessment of technical skills, VR – virtual reality
